# Full component analysis of *Tianma*-*Gouteng*-*Yin*

**DOI:** 10.1186/s13020-016-0115-8

**Published:** 2016-09-29

**Authors:** Ying-Yu Huang, Liang-Feng Liu, Rui-Qi Yue, Jun Xu, Alan Ho, Min Li, Quan-Bin Han

**Affiliations:** 1 School of Chinese Medicine, Hong Kong Baptist University, Hong Kong, China; 2 Mr. & Mrs. Ko Chi Ming Centre for Parkinson’s Disease Research (CPDR), Hong Kong Baptist University, Kowloon Tong, Hong Kong SAR

## Abstract

**Background:**

*Tianma*-*Gouteng*-*Yin* (TGY), which is common Chinese medicine formulation consisting of 11 different herbs and being used in China for the treatment of Parkinson’s disease, inflammatory conditions and cardiovascular diseases, was selected for full component analysis. The aim of this study was to quantitatively analyze the chemical profiles of ten commercial TGY samples and one sample produced in our laboratory.

**Methods:**

Ultra-high performance liquid chromatography (UHPLC) coupled with quadrupole-tandem time-of-flight mass spectrometry (Q-TOF-MS) was used to analyze the non-saccharide small molecule components of the different TGY samples. The established method was validated in terms of its linearity, sensitivity, precision, accuracy and stability. High performance liquid chromatography coupled with evaporative light scattering detection (HPLC-ELSD) was also used to quantify three major saccharides (fructose, glucose and sucrose).

**Results:**

The relative standard deviations for the precision, repeatability and stability of these compounds were less than 5 %, while the accuracy of the method was 95–105 %. Twenty-eight of the compounds found in TGY were successfully identified, with 20 being quantified. The macromolecules present in these samples were also identified using an ethanol precipitation method, representing 294.68–696.64 mg/g of the total material depending on the batch. Notably, the components identified using this method represented up to 78 % of the total weight of the TGY samples.

**Conclusions:**

The developed UHPLC/Q-TOF-MS and HPLC-ELSD methods successfully identified 28 of the complex compounds found in TGY.

## Background

*Tianma*-*Gouteng*-*Yin* (TGY) is commonly used in Chinese medicine for calming liver wind, clearing heat, promoting blood flow and nourishing the liver and kidney [1]. Several clinical studies [2–4, 12] showed that TGY can reduce the motor fluctuations of Parkinson’s disease (PD) patients. Pharmacological studies showed that TGY can prevent the apoptosis of dopaminergic neurons [2]. Furthermore, some of the active compounds isolated from the constituent of TGY exhibit neuroprotective, anti-inflammatory and cardiovascular protective effects [5–11]. In particular, the results of our recent studies revealed that some of the active compounds isolated from *Uncaria rhynchophylla* (*Gouteng*) and *Scutellaria baicalensis* (*Huangqin*), which are two of the key herbs used in TGY, promoted the clearance of pathogenic proteins and suppressed the progression of PD [5, 6, 10].

Although the clinical and pharmacological evaluation of TGY and its component herbs have been attracting interest in relevant fields, the quality analysis of TGY remains unsatisfactory. TGY consists of 11 herbs, including *Gastrodia elata* (*Tianma*), *U. rhynchophylla (Gouteng)*, *Cyathula officinalis (Chuan Niu Xi), Gardenia jasminoides (Zhizi)*, *S. baicalensis (Huangqin)*, *Eucommia ulmoides (Du Zhong)*, *Leonurus japonicus (Yimucao)*, *Taxillus chinensis (Sang Ji Sheng)*, *Polygonum multiflorum (Ye Jiao Teng)*, *Poria cocos (Fuling)* and *Haliotis diversicolor (Shi Jue Ming)* [1]. TGY contains a complex mixture of chemical ingredients, including numerous small molecules (e.g., glycosides, quinic acids, terpenes and flavonoids) and a large amount of carbohydrates (e.g., monosaccharides, oligosaccharides and polysaccharides). The only small molecules listed as quality control markers for TGY in the Chinese Pharmacopeia are baicalin and gastrodin [1]. Several methods have been reported for the analysis of a few marker compounds in TGY, including geniposide, rhynchophylline and isorhynchophylline by thin layer chromatography (TLC) and high performance liquid chromatography (HPLC) [13–15]. However, these methods found that the contents of these markers were only 2.05 %, suggesting that a large number of the compounds in TGY were being left undetermined. As a product of decoction, TGY also contains several other constituents such as macromolecules, including a large amount of carbohydrates (e.g., monosaccharides, oligosaccharides and polysaccharides) [16]. However, the analytical methods published to date for the analysis of TGY have missed this critical part. The existing analytical methods for the quality control of TGY are therefore not suitable to deliver consistent quality control.

The aim of this study was to develop a quantitative analytical method for evaluating the chemical profiles of ten commercial TGY samples and one sample produced in our laboratory. Ultra-high performance liquid chromatography (UHPLC) coupled with quadrupole-tandem time-of-flight mass spectrometry (Q-TOF-MS) was used to analyze 17 non-saccharide compounds, and high performance liquid chromatography coupled with an evaporative light scattering detector (HPLC-ELSD) was used to quantitatively determine three major saccharides. The macromolecular contents of these samples (i.e., polysaccharides, proteins and nucleic acids) were also determined by the precipitation of these molecules with ethanol.

## Methods

### Reagents, chemicals and materials

Acetonitrile (MS grade) was purchased from RCI Lab scan Ltd (Bangkok, Thailand). Analytical grade formic acid was purchased from Sigma-Aldrich (St. Louis, MO, USA). HPLC grade methanol and ethanol were obtained from Merck (Darmstadt, Germany). Deionized water was prepared using a Millipore MilliQ-Plus water purification system (Millipore, Bedford, MA, USA).

**Fig. 1:**
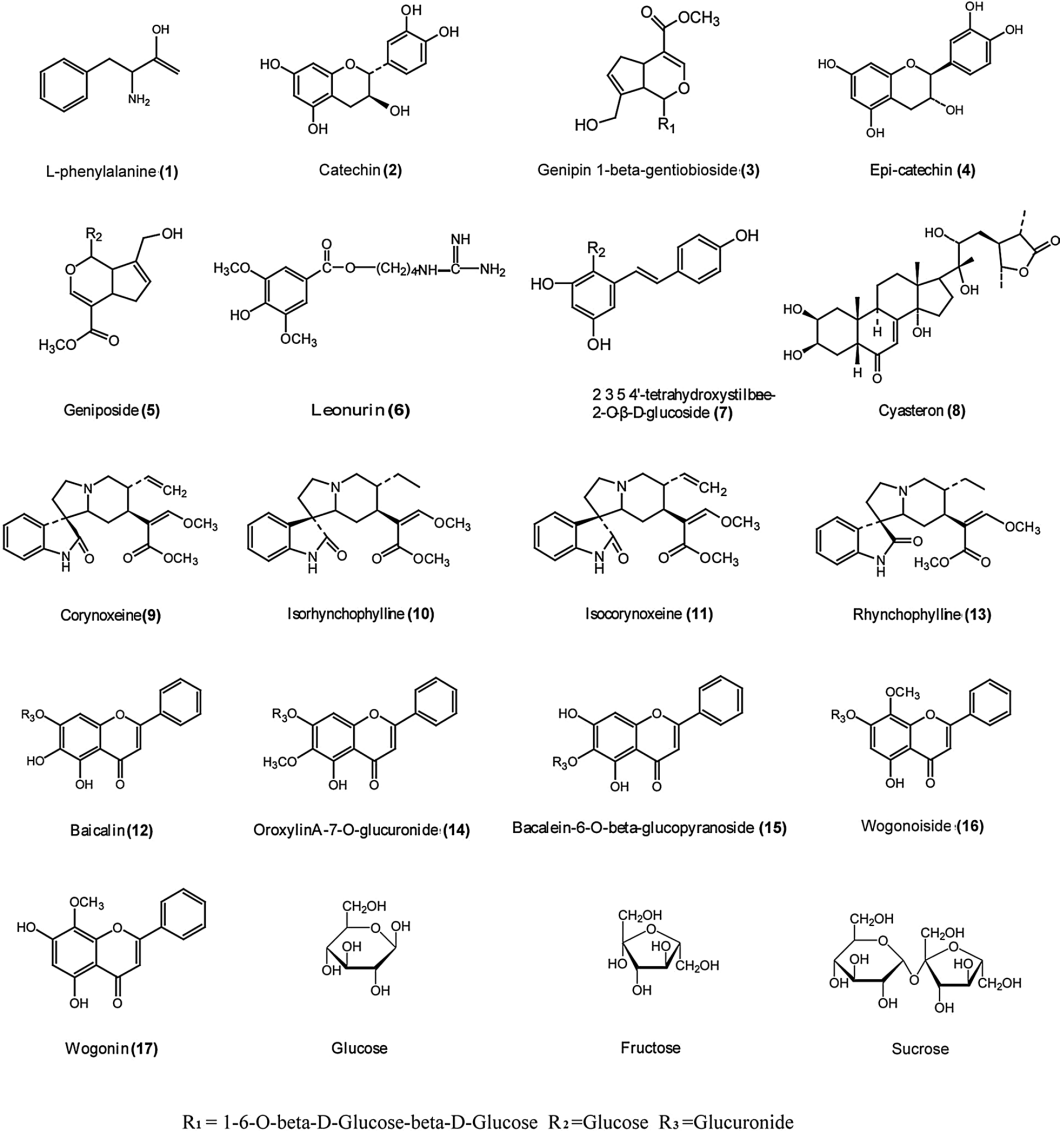
Chemical structures of the positively identified constituents of the TGY decoction

Reference standards of l-phenylalanine (**1**), catechin (**2**), genipin-1-gentiobioside (**3**), epicatechin (**4**), geniposide (**5**), leonurine (**6**), 2,3,5,4′-tetrahydroxystilbene-2-*O*-β-d-glucoside (**7**), cyasteron (**8**), corynoxeine (**9**), isorhynchophylline (**10**), isocorynoxeine (**11**), baicalin (**12**), rhynchophylline (**13**), oroxylinA-7-*O*-glucuronide (**14**), bacalein-6-*O*-beta-glucopyranoside (**15**), wogonoside (**16**) and wogonin (**17**) were purchased from Chengdu Preferred Biotechnology Co., Ltd (Chengdu, China). The identities of the reference standards were confirmed by mass spectrometry prior to being used. The purities of the reference standards were determined to be greater than 98 % by UPLC-DAD analysis based on peak area normalization. Reference standards of fructose, glucose and sucrose were purchased from Sigma-Aldrich. The structures of the reference standards are shown in Fig. 1.

Commercial samples (TGY1–10) were purchased from different pharmacies in several different regions of China, including Jilin, Beijing, Nanchang, Shenzhen and Hong Kong. The sample designated TGY11 evaluated in the current study was prepared in our laboratory using herbal materials purchased from the Mr & Mrs Chan Hon Yin Chinese Medicine Specialty Clinic and Good Clinical Practice Centre, Hong Kong Baptist University, China. All of the herbs used in the current study were identified and authenticated by Professor Zhong-Zhen Zhao from the School of Chinese Medicine, Hong Kong Baptist University, China. Voucher specimens were deposited at the School of Chinese Medicine, Hong Kong Baptist University, Hong Kong, China (Collection numbers A110607, 110991, A1202151, 1300942, A121017, 131110, 140102, 140112, 130906, 130603).

### Sample solutions preparation

The commercial samples (TGY1–10) were ground into fine powders and passed through a 60–80 mesh filter. A small accurately weighed sample (20 mg) of each powder was then dissolved in distilled water in a 10 mL volumetric flask at room temperature. All of the resulting sample solutions were filtered through a 0.22 µm nylon-membrane filter (Millipore, Barcelona, Spain) prior to being analyzed to determine the 17 non-saccharide small molecules.

The TGY11 material prepared in our laboratory consisted of the following materials: *G. elata* (*Tianma*) (9 g), *U. rhynchophylla* (*Gouteng*) (12 g), *C. officinalis* (*Chuan Niu Xi*) (18 g), *G. jasminoides* (*Zhizi*) (9 g), *S. baicalensis* (*Huangqin*) (9 g), *C. officinalis* (*Chuan Niu Xi*) (9 g), *E. ulmoides* (*Du Zhong*) (9 g), *L. japonicus* (*Yimucao*) (9 g), *T. chinensis* (*Sang Ji Sheng*) (9 g), *P. multiflorum* (*Ye Jiao Teng*) (9 g) and *P. cocos* (*Fuling*) (9 g) [1]. All of the crude materials were powdered and extracted three times by refluxing in boiling water (1:10, w/v) for 1 h. The combined extracts were then filtered and evaporated under reduced pressure, before being freeze-dried to give the TGY11 material as a powder. This powders were then prepared according to the procedure described above for the preparation of the commercial samples.

For the analysis of the saccharides, a small accurately weighed portion (10 mg) of each sample was dissolved in 2 mL of water. Ethanol (8 mL) was then added slowly added to each aqueous solution to precipitate any macromolecular components. The resulting mixtures were held for 24 h at room temperature, before being centrifuged on a 5804 Eppendorf multi-purpose centrifuge (Eppendorf Bio Tools, Radnor, PA, USA) at 3250×*g* for 15 min. The supernatant was collected and evaporated to dryness to give a residue, which was dissolved in a 1:1 (v/v) mixture of ACN and water (1 mL) and filtered through a 0.22 nylon-membrane filter (Millipore). The resulting filtrates were subjected to HPLC-ELSD analysis to determine their fructose, glucose and sucrose levels. The precipitate from the centrifugation step was dried and weighed to determine the macromolecular content of each sample [17].

### Standard solutions preparation

Reference standards of l-phenylalanine, catechin, genipin-1-gentiobioside, epicatechin, geniposide, leonurine, 2,3,5,4′-tetrahydroxystilbene-2-*O*-β-d-glucoside, cyasteron, corynoxeine, isorhynchophylline, isocorynoxeine, rhynchophylline, baicalin, oroxylinA-7-*O*-glucuronide, wogonoside, baicalein and wogonin were weighed and dissolved in different volumes of methanol containing distilled water to prepare stock solutions. Samples of these stock solutions were then mixed together to prepare a mixed standard solution. The standard saccharide solutions were prepared according to the methods described in our previous publication [18]. Reference markers of fructose, glucose and sucrose were accurately weighed and dissolved in a 1:1 (v/v) mixture of ACN and water. Calibration curves were obtained by the appropriate dilution of these mixed standard solutions.

### Analytical methods

Chromatographic analysis was performed on an Agilent 1290 UHPLC system (Agilent Technologies, Santa Clara, CA, USA) equipped with a binary pump, a thermostat-controlled column compartment, an auto sampler and a DAD detector. Separations were conducted over an Acquity UPLC BEH C18 column (2.1 × 100 mm, 1.7 µm, Waters, Milford, CT, USA) at 40 °C with a gradient elution consisting of 0.1 % formic acid in water (mobile phase A) and 0.1 % formic acid in ACN (mobile phase B). The column was eluted with the following gradient program: 0–3 min, 2 % B; 3–9 min, 2–12 % B; 9–24 min, 12–32 % B; 24–29 min, 32–75 % B; 29–29.1 min, 75–100 % B; 29.1–32 min, 100 % B. The flow rate was set at 0.4 mL/min.

An Agilent 6540 Q-TOF mass spectrometer (Agilent Technologies) equipped with a jet stream electrospray ionization (ESI) source was used to acquire the MS and MS/MS data in the positive and negative ionization modes. Data acquisition was controlled using MassHunterB.03 software (Agilent Technologies, Wilmington, USA). The operating parameters were set as follows: nebulizing gas (N_2_) flow rate, 8.0 L/min; nebulizing gas temperature, 300 °C; jet stream gas flow, 9 L/min; sheath gas temperature, 350 °C; nebulizer gas pressure, 45 psi; capillary voltages, 3000 V; skimmer voltage, 65 V; Oct RFV, 600 V; fragment voltage, 150 V. Mass spectra were recorded for *m/z* values in the range of 100–1700 with accurate mass measurements for all of the mass ions. The peak areas determined in the extracted ion chromatograms were placed into calibration curves, which were prepared by plotting the peak areas of samples containing different concentrations to calculate the non-saccharide small molecule contents contained in this decoction.

The HPLC-ELSD conditions used for the determination of the saccharides were described in our previous publication [18]. An Agilent 1100 liquid chromatograph system (Agilent Technologies, Palo Alto, CA, USA) equipped with an Alltech 2000 evaporative light-scattering detector (Grace, Deerfield, MA, USA) was used. The chromatographic separations were performed over an Asahipak NH2P-50 4E column (4.6 × 250 mm, Shodex, Tokyo, Japan) at a column temperature of 30 °C. The column was eluted with a mixture of water (mobile phase A) and ACN (mobile phase B) at a flow rate of 0.8 mL/min. The elution conditions were as follows: 0–16 min, 78 % B; 16–20 min, 78–62 % B; 20–30 min, 62–60 % B. The drift tube temperature of the ELSD was set at 120 °C, and the nitrogen flow rate was set at 3.2 L/min. The peak areas in the ELSD chromatograms were collected to calculate the concentrations of the different components. Calibration curves were generated by plotting the logarithmic values of the different peak areas against the logarithmic values of the corresponding concentrations.

### Method validation

The optimum method for the quantitative analysis of the samples was validated in terms of its linearity, sensitivity, precision, accuracy and stability. Stock solutions of the mixed standards were diluted to a variety of different concentrations to allow for the construction of calibration curves. At least six concentrations of each reference standard were analyzed in triplicate. The calibration curves were constructed by plotting the peak areas versus the concentrations of the corresponding constituents. The limit of detection (LOD) and limit of quantification (LOQ) values for the optimum conditions were determined at signal-to-noise ratios (S/N) of 3 and 10, respectively. The intra- and inter-day variations were used to evaluate the precision of our newly developed method. Six independently prepared solutions of TGY11 were analyzed within 1 day to evaluate the intra-day variability of the optimum method. To evaluate the inter-day variability of this method, we examined the same sample twice a day over 3 consecutive days. Variations were expressed as relative standard deviations (RSDs) of the data, which were calculated using the following formula: ${\text{RSD }}\left( \% \right) = \left( {{\text{standard deviation}}/{\text{mean}}} \right)\, \times 100\, \%$. A recovery test was performed to evaluate the accuracy of the optimum method by adding three different concentrations of a standard solution (i.e., low, medium and high) to TGY11, which contained known quantities of the target compounds. These samples were then analyzed in parallel using our newly established method. Each experiment was conducted in triplicate at each level. The spike recoveries were calculated using the following equation:

$$ Spike \, recovery \,  \left( \% \right)= (total \, amount \, detected\,{-}\,amount \, original)/amount \, spiked \times 100 \,\% $$

The stability of the optimal method was evaluated by analyzing the TGY11 extracts over periods of 0, 2, 4, 8, 12 and 24 h. The RSDs of the peak areas of each compound were used as an indication of the stability.

## Results and discussion

### Optimization of the chromatographic conditions

**Fig. 2:**
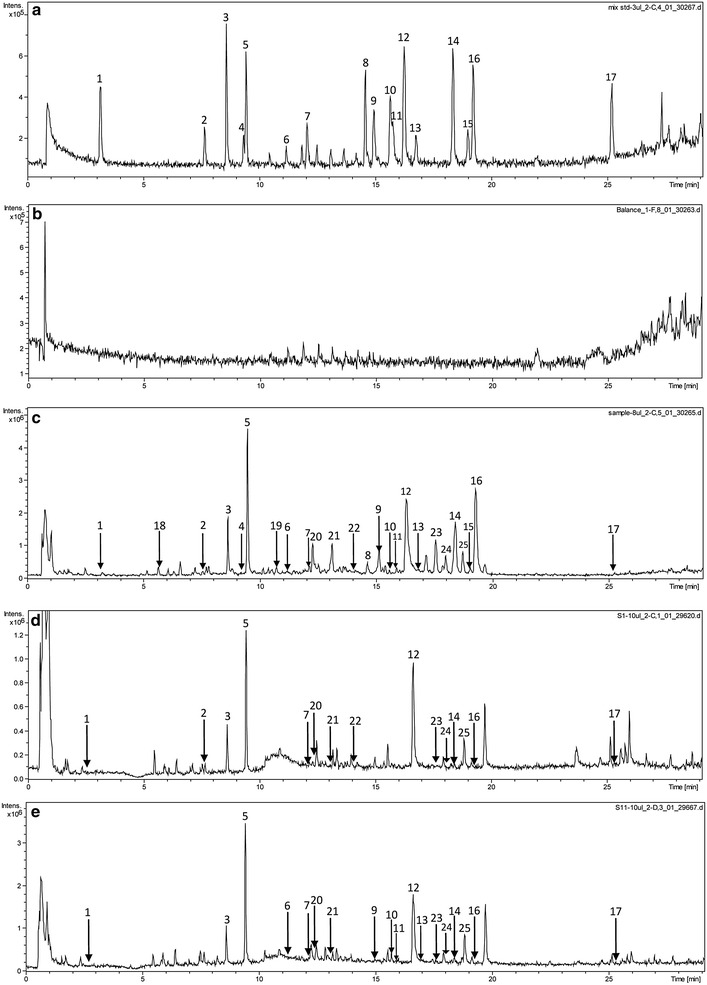
Typical UPLC-Q-TOF chromatograms. Reference standards (**a**); blank solvent (**b**); self-made sample TGY11 (**c**); Commercial samples TGY1 (**d**) and TGY6 (**e**)

The MS experiments were performed on an LC–MS system equipped with an ESI source to optimize the resolution, sensitivity and analytical time of this method. A full MS scan was obtained in the form of a total ion chromatogram (TIC). With regard to MS conditions, all of the samples were simultaneously evaluated in the positive and negative ionization modes. The positive ionization mode provided much more ESI information than the negative ionization mode and was therefore selected for the subsequent experiments. The gradient elution program was also optimized eluting with 0.1 % formic acid in water (mobile phase A) and 0.1 % formic acid in ACN (mobile phase B) under the following conditions: 0–3 min, 2 % B; 3–9 min, 2–12 % B; 9–24 min, 12–32 % B; 24–29 min, 32–75 % B; 29–29.1 min, 75–100 % B; 29.1–32 min, 100 % B. These conditions provided the best separation and peak shapes for all of compounds evaluated in the current study. Formic acid was added to the mobile phase to give a final concentration of 0.1 % to allow for the complete ionization of TGY. Representative chromatograms of the reference standards and TGY samples are shown in Fig. 2.

### Identification of chemical compounds

**Fig. 3:**
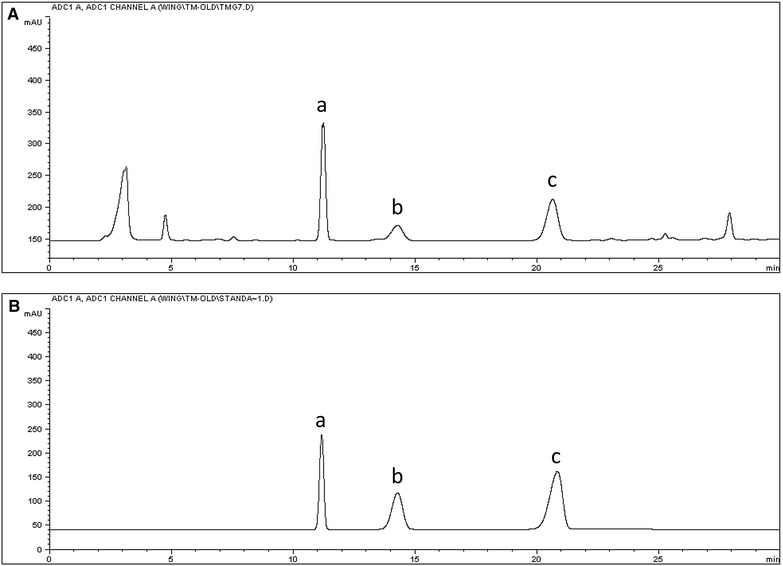
Typical HPLC-ELSD chromatograms. TGY sample (TGY11) (**A**); reference standards (**B**) (*a*: fructose, *b*: glucose, *c*: sucrose)

The peaks observed in the chromatograms of the TGY samples were primarily identified based on a comparison of their accurate molecular mass data with those of the reference standards and data from the literature [19–23]. In this way, we were able to identify 28 compounds. Data for the MS/MS fragments of the different peaks were also obtained and these results are summarized in Table 1. Representative TICs of TGY in the positive ionization mode are shown in Fig. 2a. Chromatograms of the mono- and oligosaccharides found in the different TGY samples were obtained by HPLC-ELSD for the analysis of the saccharides, and the results are shown in Fig. 3. Three peaks were identified following this analysis, including fructose, glucose and sucrose by comparing their retention times with those of the reference standards.

**Table 1:** Chromatographic and MS data (positive mode) of the constituents identified using our newly developed UPLC-Q-TOF-MS method

	R.T. (min)	Mass		Error (ppm)	Fragment ions (m/z)	Molecular formula	Identification	Herbal origin	Confirmed with reference standard
		Observed	Calculated						
1	2.80	166.0871	166.0863	4.8	120.0792 [M+H–COOH]^+^	C_9_H_11_NO_2_	l-Phenylalanine	*H. diversicolor*	Yes
2	7.50	291.0862	291.0863	5.3	139.0396 [M+H–C_8_H_7_O_3_]^+^, 123.0434 [M+H–C_8_H_7_O_4_]^+^	C_15_H_14_O_6_	Catechin	*U. rhynchophylla*	Yes
3	8.59	551.2007	551.1970	4.3	573.2007 [M+Na]^+^, 227.0753 [M+H–maltose]^+^ 209.0656 [M+H–maltose–OH]^+^	C_23_H_34_O_15_	Genipin 1-beta-gentiobioside	*G. jasminoides*	Yes
4	9.30	291.0866	291.0863	1.0	139.0381 [M+H–C_8_H_7_O_3_]^+^, 123.0422 [M+H–C_8_H_7_O_4_]^+^	C_15_H_14_O_6_	Epi-catechin	*U. rhynchophylla*	Yes
5	9.40	389.1452	389.1442	2.6	411.0792 [M+Na]^+^, 231.0366 [M+H–glucose–2H]^+^	C_17_H_24_O_10_	Geniposide	*G. jasminoides*	Yes
6	11.2	312.1554	312.1554	1.6	181.0498 [M–C_5_H_13_N_3_O], 132.1125 [M+H–C_9_H_9_N_4_O]^+^	C_14_H_22_N_3_O_5_	Leonurine	*L. japonicus*	Yes
7	12.3	407.1337	407.1351	3.4	245.0768 [M+H–glucose]^+^	C_20_H_22_O_9_	2 3 5 4′-tetrahydroxystilbene-2-*O*-β-d-glucoside	*P. multiflorum*	Yes
8	14.9	521.3146	521.3109	5.2	503.2947 [M+H–H_2_O]^+^, 485.2896 [M+H–2H_2_O]^+^	C_29_H_44_O_8_	Cyasteron	*C. officinalis*	Yes
9	15	383.1978	383.1965	3.4	383.1978[M+H]^+^	C_22_H_26_N_2_O_4_	Corynoxeine	*U. rhynchophylla*	Yes
10	15.8	385.2131	385.2122	2.3	241.1337	C_22_H_28_N_2_O_4_	Isorhynchophylline	*U. rhynchophylla*	Yes
11	15.9	383.1976	383.1965	2.9	383.1976[M+H]^+^	C_22_H_26_N_2_O_4_	Isocorynoxeine	*U. rhynchophylla*	Yes
12	16.7	447.0944	447.0922	4.9	271.0621 [M+H–glucuronide]^+^	C_21_H_18_O_11_	Baicalin	*S. baicalensis*	Yes
13	16.9	385.2126	385.2122	1.0	353.1854 [M+H–OCH_3_]^+^, 160.0754	C_22_H_28_N_2_O_4_	Rhynchophylline	*U. rhynchophylla*	Yes
14	18.9	461.1110	461.1078	5.1	285.0762 [M+H–glucuronide]^+^, 270.0507 [M+H–glucuronide–CH_3_]^+^	C_22_H_20_O_11_	OroxylinA-7-*O*-glucuronide	*S. baicalensis*	Yes
15	19.6	447.0951	447.0922	3.7	271.0601[M+H–glucuronide]^+^	C_21_H_18_O_11_	Bacalein-6-*O*-beta-glucopyranoside	*S. baicalensis*	Yes
16	19.8	461.1105	461.1078	5.9	285.0754 [M+H–glucuronide]^+^	C_22_H_20_O_11_	Wogonoside	*S. baicalensis*	Yes
17	25.6	285.0773	285.0757	5.6	270.0535 [M+H–CH_3_]^+^	C_16_H_12_O_5_	Wogonin	*S. baicalensis*	Yes
18	5.6	153.0546	153.0507	4.9	175.0380 [M+Na]^+^	C_8_H_8_O_3_	Vanillin	*G. elata*	Not yet
19	10.9	683.2537	683.2507	4.4	705.2363 [M+Na]^+^	C_32_H_42_O_16_	Pinoresinol diglucoside	*E. ulmoides*	Not yet
20	12.2	549.1646	549.1616	5.5	531.1493 [M+H–H_2_O]^+^	C_26_H_28_O_13_	Chrysin-6-C-ara-8-C-glu	*S. baicalensis*	Not yet
21	13.1	549.1620	549.1616	0.7	531.1482 [M+H–H_2_O]^+^	C_26_H_28_O_13_	Chrysin-6-C-glu-8-C-ara	*S. baicalensis*	Not yet
22	14.0	449.1143	449.1178	5.4	303.0486 [M+H–rhamnose]^+^	C_21_H_20_O_11_	Quercitrin	*L. japonicus*	Not yet
23	18.5	447.0946	447.0922	5.4	271.0617 [M+H–glucuronide]^+^	C_21_H_19_O_11_	Norwogonin-7-*O*-glucuronide	*S. baicalensis*	Not yet
24	18.9	477.1065	447.1028	4.9	301.0719 [M+H–glucuronide]^+^	C_22_H_20_O_12_	5,7,8,-Trihydroxy-6-methoxyflavone-7-glucuronide	*S. baicalensis*	Not yet
25	19.8	477.1058	477.1028	3.7	301.0718 [M+H–glucuronide]^+^	C_22_H_20_O_12_	5,6,7,-Trihydroxy-8-methoxyflavone-7-glucuronide	*S. baicalensis*	Not yet

A comparison of the retention time and MS/MS fragmentation data for the TGY samples with those of the reference standards led to the successful identification of 17 non-saccharide compounds. Specifically, peaks 1–17 were unambiguously identified as l-phenylalanine, catechin, genipin-1-gentiobioside, epicatechin, geniposide, leonurine, 2,3,5,4′-tetrahydroxystilbene-2-*O*-β-d-glucoside, cyasteron, corynoxeine, isorhynchophylline, isocorynoxeine, rhynchophylline, baicalin, oroxylinA-7-*O*-glucuronide, wogonoside, baicalein and wogonin, respectively. The chemical structures of these compounds are shown in Fig. 1.

Based on a comparison with data from the literature, peaks 18–25 were tentatively attributed to vanillin, pinoresinol diglucoside, chrysin-6-C-ara-8-C-glu, chrysin-6-C-glu-8-C-ara, quercitrin, norwogonin-7-*O*-glucuronide, 5,7,8-trihydroxy-6-methoxyflavone-7-glucuronide and 5,6,7-trihydroxy-8-methoxyflavone-7-glucuronide, respectively.

Peak 18 was determined to be vanillin based on its mass spectrum, which contained a molecular ion peak with an *m/z* value of 153.0546 for [M+Na]^+^ [24]. The mass spectrum for peak 19 contained a molecular ion peak with an *m/z* value of 705.2367 for [M+Na]^+^, which corresponded well with pinoresinol diglucoside [20, 25]. The mass spectra of peaks 20 and 21 contained molecular ion peaks with *m/z* values of 549.1646 and 549.1620, respectively, for [M+H]^+^. These peaks could be attributed to chrysin-6-C-ara-8-C-glu or chrysin-6-C-glu-8-C-ara. Although the MS/MS fragmentation spectra of these peaks contained identical signals with *m/z* values of 489, 459 and 429, the intensities of these ions varied considerably for the different peaks. Data from the literature suggested that chrysin-6-C-ara-8-C-glu would have a shorter retention time than chrysin-6-C-glu-8-C-ara. Based on this information, peak 20 was tentatively attributed to chrysin-6-C-ara-8-C-glu, whereas peak 21 was attributed to chrysin-6-C-glu-8-C-ara [21, 26]. Peak 22 was attributed to quercitrin based on a comparison of its MS and MS/MS fragmentation spectra with those reported in the literature [20]. It is noteworthy that the mass spectrum for quercitrin contained a molecular ion with an *m/z* value of 449.1178 for [M+H]^+^, whereas the MS/MS fragmentation spectrum contained a peak with an *m/z* value of 303.0486. This new peak was attributed to the formation of a quercetin aglycone fragment following the loss of a fragment with a molecular weight of 176. The ESI analysis of peak 23 in the positive ionization mode gave a molecular ion with an *m/z* value of 447.0922 for [M+H]^+^, along with a fragment ion with an *m/z* value of 271.0617 for the aglycone cation. Based on these data, peak 23 was tentatively identified as norwogonin-7-*O*-glucuronide [27]. Peaks 24 and 25 were attributed to isomeric compounds and eluted with retention times of 18.9 and 19.8 min, respectively. Furthermore, these peaks gave molecular ions with *m/z* values of 477.1065 and 447.1058, respectively. The MS/MS fragmentation spectra of these peaks both produced an ion with an *m/z* value 301. The loss of a fragment with a molecular weight of 176 in the MS/MS fragmentation spectrum was consistent with the presence of a glucuronide group. Peaks 24 and 25 were therefore tentatively attributed to 5,7,8-trihydroxy-6-methoxyflavone-7-glucuronide and 5,6,7-trihydroxy-8-methoxyflavone-7-glucuronide, respectively, based on their accurate mass and MS/MS fragmentation data [27].

### Method validation

Linearity calibration curves were constructed using at least six different concentrations of the reference standards. Each concentration of the reference standards was analyzed in duplicate. The results revealed that there was a good correlation between the concentration and peak area of these compounds, as indicated by the high coefficients of determination (r^2^ > 0.999). The linear ranges of the 17 different compounds evaluated in this way are summarized in Table 2. The LOD and LOQ values were measured using signal to noise ratios of 3:1 and 10:1, respectively, and the results are also listed in Table 2. A solution of the TGY11 material prepared in our laboratory was analyzed six times within 24 h under the optimized conditions to evaluate the intra-day variation of the method. The same solution was also analyzed on 3 successive days to assess the inter-day variation of the optimized method, as well as its precision and accuracy. The RSDs of the intra- and inter-day variations were 4.06 and 5.54 %, respectively, which indicated that our newly established method had satisfactory precision and repeatability properties. The results of spiking recovery experiments showed that our newly developed method performed well with a mean recovery in the range of 94.37–105.99 with RSDs of less than 4.5 % for the 17 positively identified compounds. The stability of our newly developed method was determined by assessing the RSDs of the peaks of the 17 positively identified compounds within 24 h. The results of this analysis revealed that the RSDs of all 17 compounds were less than 4.6 %. Taken together, these results demonstrated that our newly developed UPLC-Q-TOF method was sufficiently reliable and accurate for the simultaneous quantification of the 17 positively identified non-saccharide compounds in TGY.

**Table 2:** Results of methodology validation in terms of linear regression, LOD, LOQ, repeatability, accuracy, and stability

Analyte	Linearity			LOD (ng/mL)	LOQ (ng/mL)	Repeatability (RSD, %, n = 6)		Spike recovery (RSD, %, n = 3)			Stability (RSD, %, n = 6)
	Range (ng/mL)	Equation	r^2^			Intra-day	Inter-day	High	Middle	Low	
1	50.60–404.80	Y = 148.2X − 704.4	0.9996	13.67	45.58	3.45	4.81	96.37 (2.08)	96.55 (0.18)	105.99 (2.65)	3.16
2	397.50–3180.00	Y = 33.8X − 721.8	0.9989	104.61	348.68	2.93	4.84	98.86 (2.16)	98.67 (0.65)	94.99 (2.00)	2.46
3	329.10–10530.00	Y = 56.9X + 10454.0	0.9997	43.38	144.60	1.20	3.37	101.88 (2.21)	101.73 (0.65)	94.37 (1.72)	1.81
4	3187.50–25500.00	Y = 7.3X + 2059.8	0.9997	233.80	779.34	2.53	5.54	96.96 (2.22)	104.26 (0.78)	102.12 (5.48)	4.59
5	1659.40–53100.00	Y = 16.9X + 18376.0	0.9989	174.67	582.64	4.06	4.64	102.71 (1.99)	96.39 (0.31)	102.31 (1.94)	2.59
6	59.48–237.93	Y = 1203.4X + 9810.8	0.9992	11.65	38.84	3.78	4.66	101.38 (5.10)	104.00 (0.69)	104.50 (1.23)	3.98
7	126.28–5387.76	Y = 493.9X + 11718.0	0.9999	25.25	84.18	2.58	4.17	97.14 (2.19)	95.46 (1.27)	95.10 (0.37)	2.58
8	116.50–1864.00	Y = 170.7X + 5289.2	0.9996	17.83	59.44	1.05	4.54	96.77 (0.99)	101.81 (0.77)	104.52 (0.51)	1.56
9	13.02–208.33	Y = 378.9X + 1346.2	0.9994	2.77	9.24	1.55	3.66	101.45 (4.45)	97.26 (1.42)	104.54 (2.29)	1.29
10	13.27–106.12	Y = 12974.0X − 33626.0	0.9994	2.14	7.13	1.19	4.71	101.76 (2.68)	103.99 (1.79)	105.29 (1.15)	1.51
11	52.95–211.80	Y = 592.1X + 6742.6	0.9993	9.57	31.05	2.42	3.97	102.07 (1.53)	95.27 (0.78)	105.62 (0.58)	2.37
12	1273.44–81500.00	Y = 44.0X + 62221.0	0.9996	49.97	166.57	2.50	4.59	100.78 (2.65)	98.85 (3.36)	101.55 (3.70)	4.67
13	33.04–198.21	Y = 2767.1X − 52144.0	0.9988	7.62	25.41	3.46	4.43	95.89 (0.71)	99.58 (3.47)	94.63 (0.77)	2.85
14	204.10–13062.50	Y = 132.0X + 17621.0	0.9992	28.98	96.62	1.69	4.89	97.72 (3.65)	98.26 (0.72)	99.54 (3.98)	4.28
15	259.38–4150.00	Y = 47.4X − 1173.4	0.9989	71.38	237.95	2.64	3.89	102.64 (2.18)	99.14 (0.64)	106.11 (1.39)	2.29
16	1767.19–28275.00	Y = 117.5X + 127121.0	0.9989	26.61	88.71	1.89	4.29	97.29 (2.79)	98.27 (0.29)	96.19 (2.40)	1.87
17	118.64–2548.47	Y = 1291.1X − 78900.0	0.9998	27.23	90.76	1.07	1.69	96.64 (1.39)	101.78 (0.77)	94.88 (1.05)	1.73

All *P* values for linear regression were <0.001

### Quantification of compounds in commercial samples

**Fig. 4:**
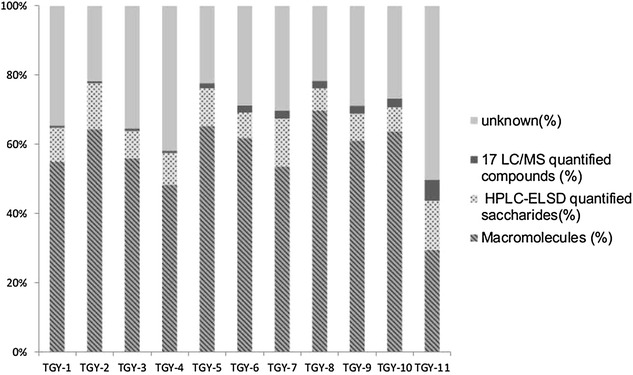
Content percentages of the chemicals determined in this study

Our newly developed UPLC-Q-TOF and HPLC-ELSD methods were successfully applied to the simultaneous determination of 20 identified compounds in 10 commercial TGY samples (TGY1–10) and one lab-prepared sample (TGY11). The results of these analyses are summarized in Table 3 and Fig. 4. The 17 positively identified non-saccharide compounds accounted for 0.5–6.1 % of the overall weight of the TGY samples, whereas the three mono- and di-saccharides accounted for 7–14.2 %. The biggest contribution to the weight of the TGY samples came from macromolecules, which account for 30–70 % of the total weight. The quantification percentage of the commercial TGY samples reached up to 78.33 %, thereby demonstrating the suitability of our newly developed method for the quality control of TGY samples in terms of accounting for most of the major components.

**Table 3:** Contents of 17 constituents in eleven batches of TMGTY samples (mg/g)

Analyte	TGY-01	TGY-02	TGY-03	TGY-04	TGY-05	TGY-06	TGY-07	TGY-08	TGY-09	TGY-10	TGY-11
1^a^	0.03^b^ ± 2.57^c^	–^d^	–	–	0.04 ± 2.50	0.08 ± 2.82	–	–	–	–	0.05 ± 3.45
2	0.39 ± 3.42	0.38 ± 2.27	0.40 ± 1.53	0.27 ± 2.72	0.25 ± 0.56	–	–	0.18 ± 1.09	–	–	0.93 ± 2.93
3	1.02 ± 1.95	0.99 ± 2.50	1.42 ± 0.13	1.27 ± 3.00	1.89 ± 2.48	1.65 ± 2.65	2.02 ± 3.16	2.54 ± 4.39	1.84 ± 0.67	2.03 ± 1.71	0.23 ± 1.20
4	–	–	–	–	–	–	–	–	–	–	4.59 ± 2.53
5	2.10 ± 3.11	2.12 ± 0.48	3.18 ± 1.90	2.92 ± 4.33	5.04 ± 4.73	5.98 ± 1.94	8.00 ± 0.63	5.69 ± 1.71	5.74 ± 2.10	6.67 ± 0.63	13.15 ± 4.06
6	–	–	–	–	–	0.05 ± 0.65	0.04 ± 1.68	0.04 ± 1.16	0.02 ± 4.77	0.02 ± 4.16	0.053 ± 3.78
7	0.03 ± 3.40	0.03 ± 0.64	0.27 ± 4.12	0.24 ± 2.86	0.09 ± 1.95	1.79 ± 1.83	1.79 ± 3.25	1.67 ± 1.96	1.72 ± 3.28	1.44 ± 2.65	0.10 ± 2.58
8	–	–	–	–	–	–	–	–	–	–	0.36 ± 1.05
9	–	–	–	–	–	0.06 ± 1.34	0.02 ± 4.10	0.02 ± 4.43	0.01 ± 1.51	0.01 ± 2.96	0.02 ± 1.55
10	–	–	–	–	–	0.03 ± 4.30	0.01 ± 1.51	0.01 ± 5.02	0.01 ± 2.85	0.01 ± 3.09	0.01 ± 1.19
11	–	–	–	–	–	0.14 ± 1.26	0.06 ± 1.13	0.04 ± 1.90	0.03 ± 2.29	0.03 ± 0.67	0.05 ± 2.42
12	1.83 ± 2.28	1.64 ± 2.29	2.08 ± 2.07	1.30 ± 4.48	5.55 ± 1.81	8.33 ± 0.95	8.18 ± 3.38	9.22 ± 1.83	10.94 ± 0.40	12.11 ± 3.41	31.34 ± 2.50
13	–	–	–	–	–	0.08 ± 1.84	0.03 ± 1.70	0.02 ± 1.13	0.03 ± 1.50	0.02 ± 1.38	0.03 ± 3.46
14	0.24 ± 4.02	0.21 ± 1.87	0.25 ± 1.41	0.22 ± 2.63	0.53 ± 0.50	0.85 ± 0.62	0.90 ± 2.66	1.02 ± 1.90	1.21 ± 1.29	1.38 ± 1.75	3.59 ± 1.69
15	–	–	–	–	–	–	–	–	–	–	0.26 ± 2.64
16	0.41 ± 3.08	0.32 ± 3.72	0.52 ± 1.53	0.44 ± 3.37	0.95 ± 1.29	1.33 ± 2.08	1.31 ± 4.36	1.60 ± 4.13	1.74 ± 0.40	2.06 ± 0.56	5.98 ± 1.89
17	0.09 ± 2.50	0.05 ± 2.12	0.06 ± 3.66	–	–	0.17 ± 0.20	0.08 ± 4.89	0.09 ± 3.89	0.11 ± 1.69	0.12 ± 3.12	0.57 ± 1.07
Fructose	38.03 ± 1.45	70.92 ± 0.99	30.26 ± 3.02	41.04 ± 1.03	45.46 ± 1.21	38.08 ± 1.66	80.56 ± 1.86	26.79 ± 1.45	40.15 ± 3.67	38.12 ± 0.71	69.60 ± 2.86
Glucose	45.70 ± 1.07	46.34 ± 0.39	34.31 ± 0.41	37.30 ± 0.50	46.35 ± 3.21	30.64 ± 4.48	52.21 ± 1.59	32.79 ± 4.38	33.07 ± 4.93	27.89 ± 2.10	67.87 ± 4.12
Sucrose	14.50 ± 0.63	14.71 ± 0.04	13.52 ± 1.02	12.92 ± 4.74	16.18 ± 4.79	4.72 ± 1.78	5.85 ± 1.23	5.01 ± 2.13	3.83 ± 4.33	4.10 ± 4.56	4.03 ± 1.02
Mac. molecular	549.36 ± 2.81	643.87 ± 4.33	559.69 ± 3.69	483.25 ± 0.48	655.19 ± 0.94	618.28 ± 0.02	535.61 ± 0.06	696.64 ± 4.11	611.02 ± 2.92	636.59. ± 0.03	294.98 ± 1.11

^a^The compound numbers are the same as in Fig. 1

^b^The data was present as average of triplicate determinations

^c^SD value of two times determination results

^d^Under the limit of quantification (LOQ)

All 17 of the positively identified non-saccharide compounds were found in the lab-prepared sample (TGY11), with baicalin being the most abundant. The contents of these compounds varied considerably in the samples collected from the different manufacturers. For example, rhynchophylline, isorhynchophylline, corynoxeine and isocorynoxeine are considered to be the four main bioactive constituents of *U. rhynchophylla* [6, 13, 28]. However, the amount of corynoxeine detected in TGY6 was six times greater than that detected in TGY9. Furthermore, none of these four alkaloids were detected in TGY1–5. This result implied that there were inconsistencies in the quality of the TGY decoctions. These variations in the quality could be attributed to various factors, including differences in the origins of the plants, as well as differences in the planting methods, harvest time, geographical climate and processing methods. The fructose, glucose and sucrose contents of the samples also fluctuated considerably between the different samples. Once again, these variations could be attributed in part to differences in the pretreatment processes or manufacturing procedures. It is noteworthy that much larger amounts of sucrose were found in TGY1–5 than any of the other samples. In terms of the macromolecular contents of these samples, the contents of TGY8 and TGY11 were 696.64 and 294.98 mg/g, respectively. This variation may have been introduced as a consequence of the difference in the raw materials, extraction process or processing procedures. Also, the presence of excipients in the sample matrix could have a significant impact on the contents of the sample being investigated.

Compared with other methods reported in the literature [1, 13–15, 29], which have been used to determine the total contents of only a few chemical markers at concentrations as low as 2.05 %, the method described herein was used to quantify almost 60–80 % of the compounds found in TGY, representing a clear majority. The percentage ratios of the components determined in the current study are shown in Fig. 4. These results show that the quantification percentages of the commercial samples could reach up to 78.33 %, whereas that of TGY11 was much lower at 50 %. Notably, more than 40 % of the chemicals present in TGY11 remained unidentified. These chemicals could include amino acids, inorganic salts or some other ingredients. However, it is also possible that the addition of 80 % ethanol was not sufficient to precipitate all of the polysaccharides completely, and that those with lower molecular weights remained in solution [17] and their content could not be determined.

## Conclusions

The developed UHPLC/Q-TOF-MS and HPLC-ELSD methods successfully identified 28 of the complex compounds found in TGY, and quantified 20 of them.

## Abbreviations

TGY: *Tianma*-*Gouteng*-*Yin*
PD: Parkinson disease
UHPLC: ultra high-performance liquid chromatography
Q-TOF-MS: quadrupole-tandem time-of-flight mass spectrometry
HPLC: high performance liquid chromatography
ELSD: evaporative light scattering detection
RSD: relative standard deviation
